# Dibenzo[*b,f*]oxepine Molecules Used in Biological Systems and Medicine

**DOI:** 10.3390/ijms241512066

**Published:** 2023-07-27

**Authors:** Hanna Krawczyk

**Affiliations:** Department of Organic Chemistry, Faculty of Chemistry, Warsaw University of Technology, Noakowskiego 3, 00-664 Warsaw, Poland; hanna.krawczyk@pw.edu.pl

**Keywords:** dibenzo[*b,f*]oxepine derivatives, synthesis, biological data, medical data

## Abstract

In this short review, including 113 references, issues related to dibenzo[*b,f*]oxepine derivatives are presented. Dibenzo[*b,f*]oxepine scaffold is an important framework in medicinal chemistry, and its derivatives occur in several medicinally relevant plants. At the same time, the structure, production, and therapeutic effects of dibenzo[*b,f*]oxepines have not been extensively discussed thus far and are presented in this review. This manuscript addresses the following issues: extracting dibenzo[*b,f*]oxepines from plants and its significance in medicine, the biosynthesis of dibenzo[*b,f*]oxepines, the active synthetic dibenzo[*b,f*]oxepine derivatives, the potential of dibenzo[*b,f*]oxepines as microtubule inhibitors, and perspective for applications of dibenzo[*b,f*]oxepine derivatives. In conclusion, this review describes studies on various structural features and pharmacological actions of dibenzo[*b,f*]oxepine derivatives.

## 1. Introduction

**Figure 1:**
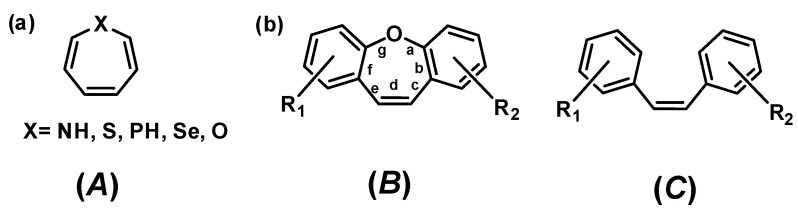
Structure of (**a**) azepine, thiepine, phosphepine, selenepine, and oxepine (**A**); (**b**) dibenzo[*b*,*f*]oxepine (**B**) (with the designation of the bonds) and stilbene with the *Z* configuration (**C**).

Seven-membered heterocycles are an important class of molecules which have found applications in medicine and biology [1,2,3,4,5]. This class includes compounds containing nitrogen, sulfur, phosphorus, selene, or oxygen atoms (etc.) and that are systematically named azepines, thiepines, phosphepines, selenepines, and oxepines, respectively, Figure 1(aA). Seven-membered oxygen heterocycles occupy a prominent place in the chemistry of natural products [1,2,3,4,5]. For example, oxepines are found among alkaloids, the best known of which are strychnine and clarine, and in the field of terpenes, several plant-derived polycyclic elactones have been described, of which bitter limonine is a well-known example. Dibenzo[*b*,*f*]oxepine belongs to this group, Figure 1(bB). Dibenzo[*b*,*f*]oxepines are compounds consisting of a heterocyclic seven-membered oxepine ring with one double bond and an oxygen heteroatom and benzene rings (with different substituents) attached at the *b* and *f* positions of the oxepine. Structurally dibenzo[*b*,*f*]oxepines are analogs of *Z*-stilbene (Figure 1(bC)). Dibenzo[*b*,*f*]oxepines are classified in the LOTUS (Natural Products Online) database as monomeric stilbenes [4].

Dibenzo[*b*,*f*]oxepine scaffold is an important framework in medicinal chemistry, and its derivatives occur in several medicinally relevant plants. These compounds are characterized by various valuable properties, such as anticancer, antihypertensive, anti-inflammatory, antidepressant, antiestrogen, antipsychotic, neuroprotective, anxiolytic, and insecticidal properties [1,2,3,4,5]. The structure, production, and therapeutic effects of dibenzo[*b*,*f*]oxepines have not been extensively discussed thus far and are presented in this review. In addition, the possibilities for the use of dibenzo[*b*,*f*]oxepine derivatives in the future are presented.

## 2. Extracting Dibenzo[*b*,*f*]oxepines from Plants and Its Significance in Medicine

An interesting branch of science is the search for active compounds extracted from plants [6]. In this way, it was possible to discover the biological effects of many molecules, including dibenzo[*b*,*f*]oxepine derivatives. This chapter presents the most known dibenzo[*b*,*f*]oxepine derivatives obtained from various plants.

### 2.1. Pacharin

**Figure 2:**
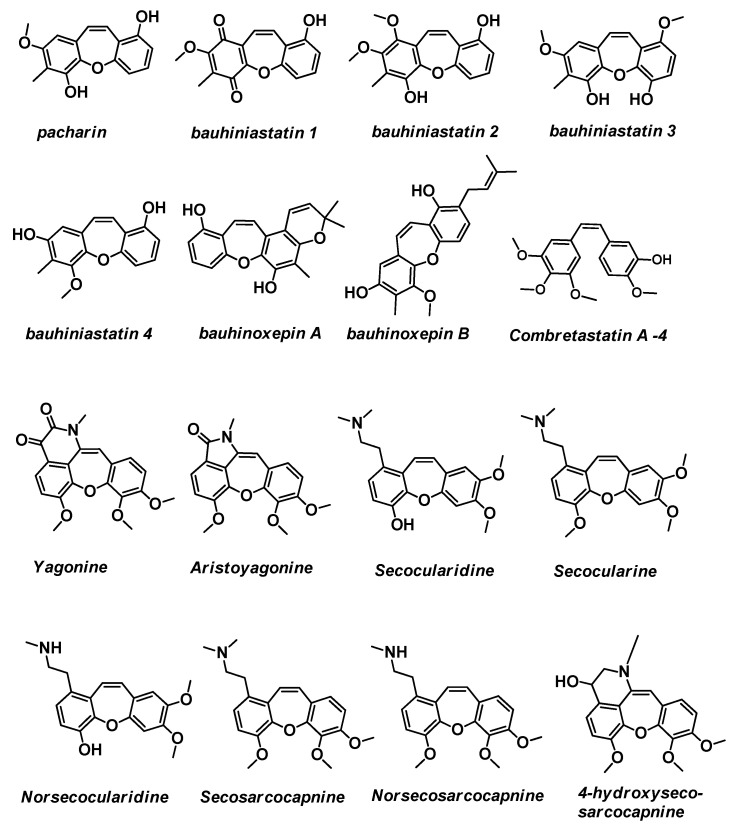
Structure of pacharin, Bauhiniastatins 1–4 and Bauhinoxepins A and B, Combretastatin A-4, Yagonine, Aristoyagonine, Secocularidine, Secocularine, Norsecocularidine, Secosarcocapnine, Norsecosarcocapnine, and 4-hydroxysecosarcocapnine.

The most commonly used, pacharin (Figure 2) has been obtained as an extract from the genus *Bauhinia* (family *Fabaceae*) of trees and shrubs, inhabiting numerous geographic locations in warm climates. The examples of its sources are as follows:

a. the stem bark, stem wood, and roots of *Bauhinia ungulate* L. [7];
b. leaves, stems, pods, and roots of *Bauhinia purpurea* [8,9];
c. the heartwood of *Bauhinia racemosa* Lamk. [10];
d. the stem bark of *Bauhinia aculeata* L. [11];
e. the *Bauhinia acuruana* Moric shrub [12,13];
f. vine stems from *Millettia dorwardi* Collet Hemsl [14];
g. the bark of *Rhamnus caroliniana* [15];
h. the flowers of *Cercis chinensis* Bunge [16].

In addition to the fact that it has been used in folk medicine for centuries to treat diabetes, infections, pain, and inflammation, its effect on various cancer cells has been studied [8,12,14]. Pacharin demonstrated an antiproliferative effect on the following cell lines: the human tumor cell lines MCF-7 (breast adenocarcinoma) IC_50_ 20 mM, lung carcinoma NCI-H292 IC_50_ 11.11 mM or NCI-H460 IC_50_ 4.2 mg/mL, HL-60 (pro-myelocytic leukemia) IC_50_ 8.15 mM, colon carcinoma HTC-116 IC_50_ 19.26 mM or KM20L2 IC_50_ > 10 mg/mL, SF-295 (glioblastoma) IC_50_ 14.44 mM, OVCAR-8 (ovarian carcinoma) IC_50_ 23.33 mM, HepG2 (hepatic cell line) IC_50_ 52.08 mM, Raji (the first continuous human cell line of hematopoietic origin) IC_50_ 40.17 mM and KG-1 (acute myeloid leukemia cell lines) IC_50_ 61.22 mM, BXPC-3 (pancreas cell line) GI_50_ 4.3 mg/mL, SF268 (CNS cell line) GI_50_ 3.1 mg/mL, and and DU-145 (prostate cancer cell lines) GI_50_ 3.3 mg/mL Pacharin was also not cytotoxic to normal human peripheral blood mononuclear cells (IC_50_ > 100 µM) [13,14,15]. Pacharin also shows larvicidal activity against *Aedes aegypti* [17,18].

### 2.2. Bauhiniastatins 1–4

The next known family are Bauhiniastatins 1–4 and also Bauhinoxepins A and B, which are bioactive natural products (Figure 2). Bauhiniastatins 1–4 were evaluated for cancer cell growth inhibition against the pancreas BXPC-3 cell line, breast MCF-7 cell line, CNS SF268 cell line, lung NCI-H460 and NCI-H292 cell line, colon KM20L2 cell line, prostate DU-145 cell line, colon carcinoma HTC-116 cell line, pro-myelocytic leukemia HL-60 cell line, glioblastoma SF-295 cell line, ovarian carcinoma, and OVCAR-8 cell line [8,12,19]. Each was found to exhibit significant inhibition against the cancer cell lines (GI_50_ (μg/mL) from 2.4 to 25.7). The authors noticed that the general structure of Bauhiniastatins is reminiscent of the Z-stilbene geometry required for strong antiangiogenesis activity. A similar construction occurs in the combretastatin series of anticancer drugs from the *Combretum caffrum* tree. For example, combretastatin A4 is a microtubule-targeting agent that binds β-tubulin with Kd of 0.4 μM (Figure 2) [20,21].

The antioxidant activity of Bauhiniastatin 4 at various concentrations (from 500 to 7.81 ppm) has been evaluated against DPPH radical scavenging [11]. It was discovered that Bauhiniastatin 4 reduces free radicals at a low concentration of about 9 ppm against DPPH radical scavenging. Bauhiniastatin 4 showed free radical scavenging activity with an IC_50_ value of 32.7 μM, which is lower than that of the used standard (positive control) ascorbic acid (IC_50_ 62.8 μM). The authors noted that the presence of hydroxyl groups at C-3 in Bauhiniastatin 4 increased antioxidant activity, while the presence of a methoxy group at C-3 (as is in pacharin) lowered antioxidant activity. Pacharin in similar studies showed no activity.

### 2.3. Bauhinoxepins A and B

In turn, Bauhinoxepins A and B (Figure 2) were isolated from the root extract of *Bauhinia saccocalyx* by Kittakoop and coworkers in 2004 [22]. This compound shows significant antimycobacterial activities, with an MIC value of 6.25 and 12.5 g/mL, respectively. 

### 2.4. Yagonine, Aristoyagonine 

Yagonine is a new oxidized isocularine alkaloid from the *Sarcocapnos Enneaphylla* plant which is native to southwestern Europe and northern Africa (Figure 2) [23]. This last compound turned out to be a bromodomain inhibitor [24]. Bromodomain-containing protein 4 (Brd4) is known to play a key role in tumorigenesis. Aristoyagonine exerted cytotoxicity in I-BET-762-sensitive cancer cells, but also I-BET-762-resistant cancer cells. This is the first report to describe the natural compound as a Brd4 bromodomain inhibitor.

### 2.5. Secocularidine, Secocularine, Norsecocularidine 

Secocularidine and Secocularine, were isolated as two members of a new group of isoquinoline-related alkaloids, the secocularines (Figure 2) [25]. Norsecocularidine was next isolated (Figure 2) [26]. It is a subfamily of the family *Papaveraceae* (the *poppy* family) native to the Northern Hemisphere and South Africa. They were also later extracted from the aerial parts of *Sarcocapnos crassifolia* subsp. *speciosa* [27]. The tested molecules showed in vitro inhibitory activity on ^3^H-dopamine uptake by rat striatal synaptosomes, with IC_50_ values 13.4 μM and 28.2 μM for Secocularidine and Secocularine, respectively [28].

### 2.6. Secosarcocapnine, Norsecosarcocapnine, 4-Hydroxysecosarcocapnine

Chemical extractions of *Sarcocapnos enneaphylla* and other *Sarcocapnos* species [29] resulted, inter alia, in the isolation of Secosarcocapnine, Norsecosarcocapnine, and 4-hydroxysecosarcocapnine [30]. They are alkaloids of the cularine group (Figure 2) [31,32]. The relaxant effects of the alkaloids of the cularine group have been studied on the guinea-pig isolated trachea and human bronchus against contractions induced by acetylcholine, histamine, neurokinin A, and KCl. Among the alkaloids tested, the most potent was the cularine group, the relaxant activity of which was between those of papaverine and theophylline. The data show that alkaloids of the cularine group display non-specific antispasmogenic activity on guinea-pig and human airways.

### 2.7. Artocarpol A, Artocarpols D-G (Figure 3)

Novel phenolic compounds Artocarpol A, Artocapol D, Artocarpol E, Artocarpol F-chiral, Artocarpol G, and Artocarpol I were isolated from the root bark of *Artocarpus rigida* by the group of S.-Z. Yang [33,34,35,36,37]. Artocarpol A inhibited superoxide formation in phorbol 12-myristate 13-acetate (PMA)-stimulated rat neutrophils in a concentration-dependent manner with an IC_50_ value of 13.7 ± 0.7 μm and also showed an inhibitory effect on tumor necrosis factor-a (TNF-a) in RAW264.7 cells (established from the preparation of macrophages of a BAB/14 mouse) [33,34,35,36,37]. In addition, this compound stimulates superoxide anion generation in rat neutrophils [38]. In turn, the anti-inflammatory activities of Artocarpol I were studied in vitro by measuring the inhibitory effect on the chemical-mediator release from mast cells, neutrophils, macrophages, and microglial cells and established that when inhibited in a concentration-dependent manner, the formyl-Met-Leu-Phe (fMLP)/cytochalasin B (CB)-stimulated superoxide anion formation in neutrophils has an IC_50_ value of 17.1 ± 0.40 μm [33,34,35,36,37].

**Figure 3:**
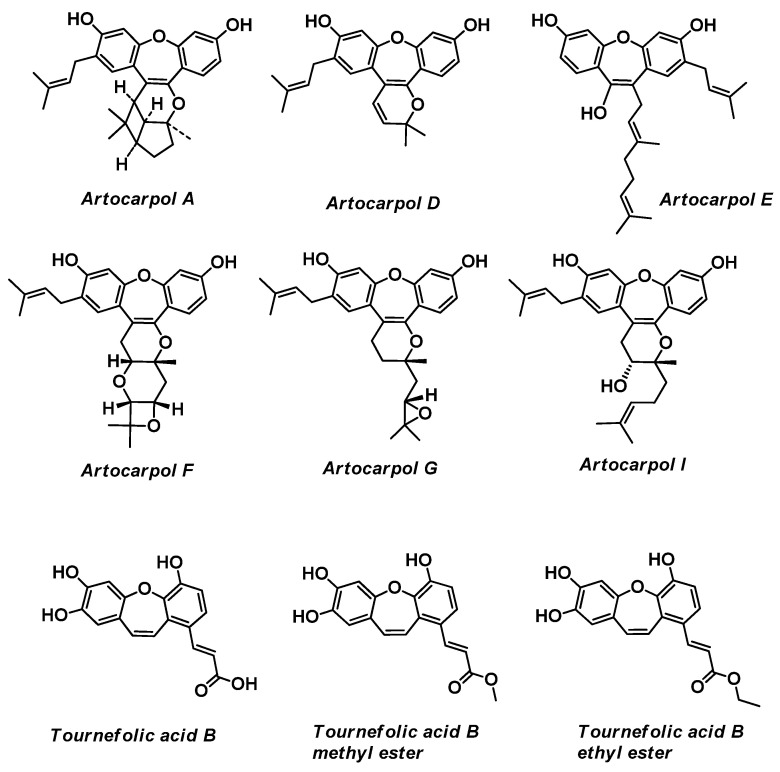
Structure of Artocarprol A, Artocarprol D, Artocarprol E, Artocarprol F, Artocarprol G, Artocarprol I, Tournefolic acid B, Tournefolic acid B methyl ester, and Tournefolic acid B ethyl ester.

### 2.8. Tournefolic Acid B, Tournefolic Acid B Methyl Ester, and Tournefolic Acid B Ethyl Ester (Figure 3)

Tournefolic acid B, Tournefolic acid B methyl ester, and Tournefolic acid B ethyl ester (TAB derivative) were isolated from the stems of *Tournefortia sarmentosa* Lam. [39,40]. *Tournefortia sarmentosa* Lam. (the family *Boraginaceae*) extract has been used in Taiwan as a detox agent, an anti-inflammatory agent, and for promoting blood circulation for the removal of blood stasis [39]. The TAB derivative effectively attenuates neurotoxicity mediated by Aβ, glutamate, NMDA, and 1-methyl-4-phenylpyridinium. Specifically, TAB achieves this by abrogating calcium overload in the mitochondria and hindering the caspase 8-truncated bid—cytochrome C pathway [41,42,43,44].

### 2.9. The Physicochemical Approaches to Estimate Medical Activity

Several physicochemical and biological properties can be associated with the activity of compounds based on the knowledge of their chemical structure. To analyze the properties of the dibenzo[*b,f*]oxepine scaffold affecting the possibility of passive penetration through biological membranes, calculations of lipophilicity (XLogP3-AA), molar mass, topological polar surface area (TPSA [Å2]), and number of hydrogen bond donors and hydrogen bond acceptors were made [45,46]. For orally administered compounds, the value of TPSA should be less than 120 Å2, and that for compounds intended to cross the blood–brain barrier should be lower than 60–70 Å2 [47], the molar mass < 500, hydrogen bond donor count < 5, and hydrogen bond acceptor count < 10. The listed parameters for dibenzo[*b*,*f*]oxepine derivatives extracted from plants are presented in Table 1. The structure of the dibenzo[*b*,*f*]oxepine scaffold meets the literature criteria for orally administered and potentially active compounds (XLogP3-AA 3.9; molar mass 194.23 g/mol; TPSA 9.2 Å2; hydrogen bond donor count is 0; and hydrogen bond acceptor count is 1). Therefore, it is not surprising that this scaffold is part of the molecules exhibiting therapeutic properties (Table 1). All extracted compounds have TPSAs less than 120 Å2, and often less than 70 Å2 for molecules intended to cross the blood–brain barrier. The molar mass is also <500 for all molecules and most chemicals’ active XLogP3-AA is <5. Notably, hydrogen bonding is a key element of molecular recognition [48]. Over the last decades, it has been found in physicochemical phenomena such as DNA base pairing, enzymatic catalysis, [49] host—guest complex formation, [50] and solid-state stabilization [51,52,53]. The parameters describing interactions of hydrogen bonds are the hydrogen bond donor/acceptor counts [45,46,48]. In the discovery set, ‘the rule of 5′ [45,46] predicts that good absorption or permeation is more likely when there are less than 5 H-bond donors and 10 H-bond acceptors. For all presented compounds, the hydrogen bond donor count is <5 and the hydrogen bond acceptor count is <10. From Table 1, it can be concluded that the rule of five is fulfilled for almost all compounds and the molecules show biological activity.

## 3. Biosynthesis of Dibenzo[*b*,*f*]oxepines

**Scheme 1:**
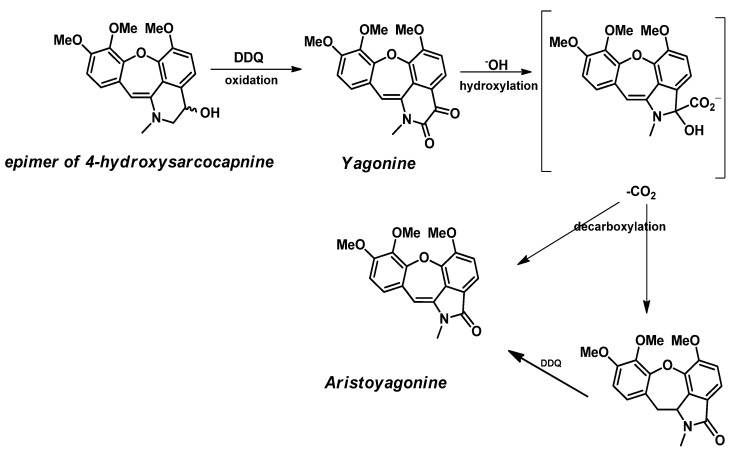
Possible biosynthetic pathways of Yagonine and Aristoyagonine.

The biosynthetic pathway of dibenzo[*b*,*f*]oxepins has not yet been thoroughly explored. However, a possible biosynthetic route for the dibenzo[*b*,*f*]oxepine derivatives Yagonine and Aristoyagonine has been proposed by the Vidal group (Scheme 1) [54]. Yagonine was obtained from the oxidation of 4-hydroxysarcocapnine epimers with a 41% yield. Aristoyagonine was received from Yagonine via dehydroxylation and decarboxylation with a low yield of 7%. The authors postulate this sequence of transformations because such transformations have previously been observed to occur, typically in other alkaloids [55].

## 4. Structure of Dibenzo[*b*,*f*]oxepine and Synthesis of Dibenzo[*b*,*f*]oxepine Derivatives

Based on the analysis of the diffraction image made with the Enraf-Nonius CAD-4 diffractogram, it was found that dibenzo[*b*,*f*]oxepine crystallizes in the orthorhombic system marked with the space group P 21/n 21/a 21/m (Pnam) [56], although depending on the substituents attached to the aromatic rings, the arrangement may differ slightly. As the authors stated “the ground-state structure” resembles that of a butterfly (saddle-shaped), with its wings highly bent backwards [57]. The optimum structures of dibenzo[*b*,*f*]oxepine were also calculated using the DFT B3LYP/6-311++G(2d,p) method and with the polarizable continuum model (PCM) (which is a commonly used method in computational chemistry to model solvation effects) [58]. Calculations have shown that the dibenzo[*b*,*f*]oxepine scaffold is not planar and that it adopts a basket conformation in solution. The dihedral angles between the aromatic rings connected with oxygen and the double bond for dibenzo[*b*,*f*]oxepines are about 64.9–68.8°. The characteristic parameters in ^1^H NMR spectra [58] for the dibenzo[*b*,*f*]oxepine scaffold are: chemical shifts for aromatic protons in the range 6.5–8.4 ppm, a coupling constant ^3^J about 7.5–8.5 Hz, chemical shifts for olefinic protons about 7 ppm, and a characteristic ^3^J coupling constant about 11 Hz for protons in the AB spin system in the *Z* configuration. In IR spectroscopy [58], bands in the range of 3080–3030 cm^−1^ can be seen coming from the valence vibrations = C-H of aromatic compounds. The characteristic vibration bands of the aromatic ring from 1625 cm^−1^ to 1440 cm^−1^ are also visible. Aromatic resonance structures of dibenzo[*b*,*f*]oxepine in the S_0_, S_1,_ and T_1_ states show that it can act as an “aromatic chameleon” compound, as the authors claim in reference [59]. 8π-Electron cyclic molecules in their S_0_ states typically adopt non-planar structures that are non-aromatic rather than antiaromatic. Dibenzo[*b*,*f*]oxepine S_0_, adopts no planar structures and takes fold conformations [60]. Furthermore, dibenzo[*b*,*f*]oxepine shows a large Stokes’ shift (λ_ex_ = 280 nm; λ_em_ = 480 nm in cyclohexane at 23 °C) and a well-defined vibrational structure in the fluorescence spectrum [57,61], evidence that a change from a no-planar to a planar conformation occurs in the S1 state. In the first excited states (S1 and T1), the dibenzo[*b*,*f*]oxepine scaffold can become planar because of the cyclically conjugated (4n + 2) π electrons of the central ring, which meet the requirements for excited-state aromaticity. Dibenz[*b*,*f*]oxepine shows an increased photostability when compared with its 10,11-dihydrogenated analog, a feature that could be related to the gain in S1-state aromaticity of a cyclic system with (4n + 2) π-electrons.

Dibenzo[*b*,*f*]oxepine was first synthesized in 1911 by Pschorr and Knöffler during the nitration of α, β-diaryl acrylic acids [62]. Since then, its scaffold has been an important framework in medicinal chemistry, and its derivatives occur in several medicinally relevant plants [63,64]. The synthesis of dibenzo[*b*,*f*]oxepines can be carried out in some ways [65]. Several methods are discussed below.

- Wagner–Meerwein rearrangement [66]

**Scheme 2:**
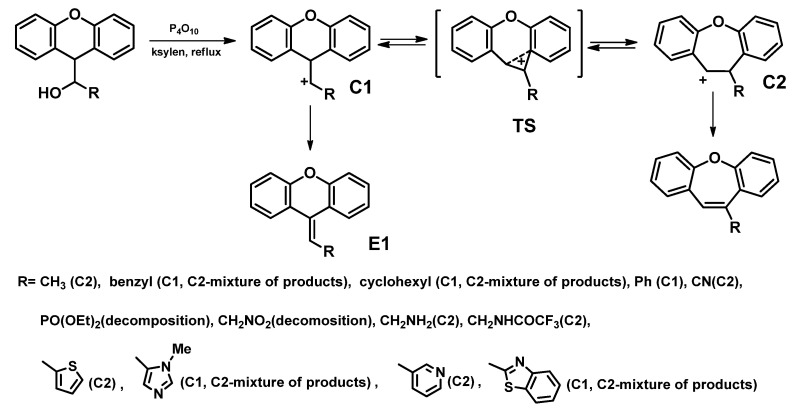
Wagner–Meerwein rearrangement on the example of 9-hydroxyalkylxanthene. Preferred carbocation in brackets.

Dibenzo[*b*,*f*]oxepines can be obtained from 9-hydroxyalkylxanthene via Wagner-Meerwein rearrangement (Scheme 2) [66]. The solvent used is xylene. This reaction involves phosphorus (V) oxide as a generating substance carbocation (C1). This carbocation can transform into a transition state (TS), which then converts to the second carbocation (C2), which in turn converts to the desired product. C1 can also undergo β-elimination to the xanthenylid-9-ene derivative (E1). The nature of the R substituent determines which of the two reactions is favored. Depending on it, the C1 or C2 carbocation is more energetically stable.

1. Ullman coupling with a subsequent Friedel–Crafts reaction [67,68]

**Scheme 3:**
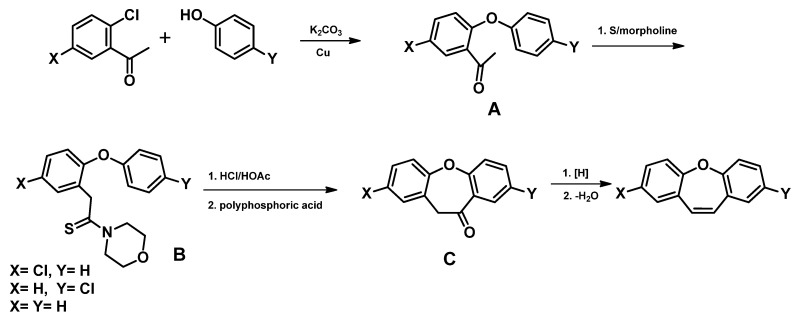
Synthesis of dibenzo[*b*,*f*]oxepines via Ullman coupling reaction followed by Friedel–Crafts reaction.

In this method, the appropriate acetophenone halogen derivative is reacted with a phenol derivative (the so-called Ullman diaryl ether synthesis, Scheme 3) [67,68], which leads to the preparation of a diphenyl ether derivative (**A**). Conversion of the ketone to acid or its derivative (**B**) then takes place, followed by a Friedel–Crafts reaction to give the compound (**C**). In the last step, the reduction of the carbonyl group is carried out with subsequent dehydration, which leads to the preparation of dibenzo[*b*,*f*]oxepine.

2. Ullmann coupling and ring-closing metathesis reaction [69,70]

**Scheme 4:**
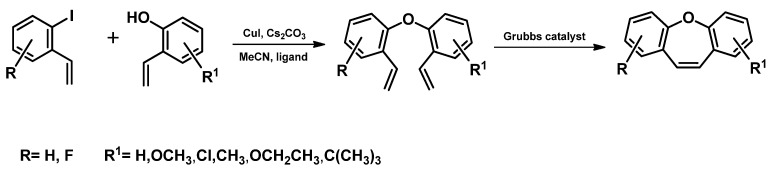
Reaction for the preparation of dibenzo[*b*,*f*]oxepines on the example of the Ullmann coupling reaction, and then ring-closing metathesis reaction.

The substrates of the Ullmann coupling reaction are the halogen derivative and hydroxy derivative of styrene. The compounds undergo the following reactions: metathesis with the closure of the ring and conversion to the dibenzo[*b,f*]oxepine derivative (see Scheme 4) [69,70].

3. Preparation from 2-halogenobenzaldehydes [71]

**Scheme 5:**
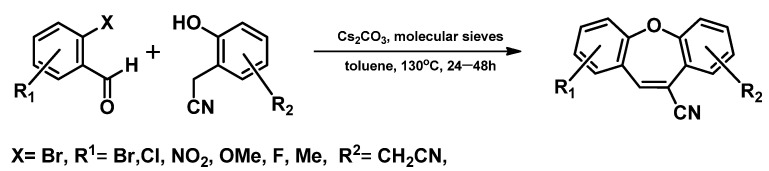
Synthesis of dibenzo[*b*,*f*]oxepines from 2-halogenobenzaldehyde and 2-(2-hydroxyphenyl) acetonitrile derivatives.

This is a one-step method that produces dibenzo[*b*,*f*]oxepine. The reaction of 2-halogenobenzaldehyde with a derivative of 2-(2-hydroxyphenyl) acetonitrile runs through sequential aldol condensation and an intramolecular ether formation reaction in the presence of Cs_2_CO_3_, molecular sieves, and in toluene as a solvent (Scheme 5) [71].

4. Intramolecular aromatic nucleophilic substitution S_N_Ar [58]

**Scheme 6:**
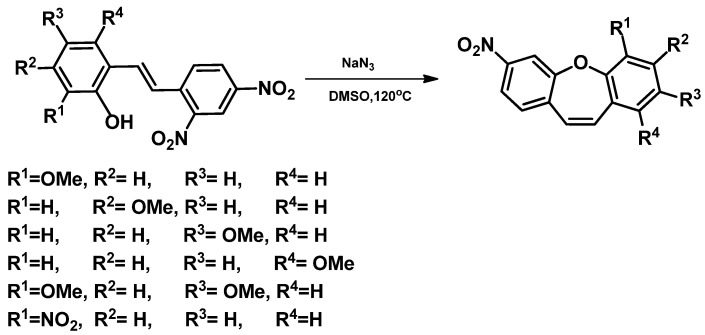
Synthesis of dibenzo[*b*,*f*]oxepines via intramolecular aromatic nucleophilic substitution S_N_Ar.

The above method of dibenzo[*b*,*f*]oxepine synthesis was developed by the research team of Krawczyk et al. [58] (Scheme 6). Intramolecular aromatic nucleophilic substitution is conducted in a sodium azide environment, which increases the yield of the reaction by about 50%. Two strongly electron-withdrawing nitro groups are attached to one of the stilbene rings. The donating hydroxyl group is located on the second ring. The substitution of one of the nitro groups by the hydroxyl group gives dibenzo[*b*,*f*]oxepine. The yield of the reaction is estimated from 88 to even 95% (Scheme 6).

5. Knoevenagel condensation [72]

**Scheme 7:**
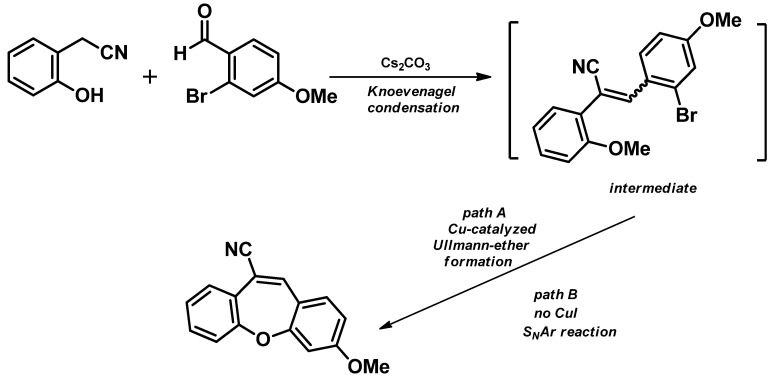
Proposed reaction mechanism of Knoevenagel condensation and, next, the Ullmann ether formation in the synthesis of dibenzo[*b*,*f*]oxepine.

The authors propose a two-step course of reaction: the first step is a Knoevenagel condensation, and the next is the Ullmann ether formation (Scheme 7) [72]. The intermediate (Scheme 7) after Knoevenagel condensation is converted to dibenzo[*b*,*f*]oxepine via cyclization. The reaction from the intermediate can run with CuI (path A) or without CuI (path B). The yield of this reaction is high at 92%.

**Scheme 8:**
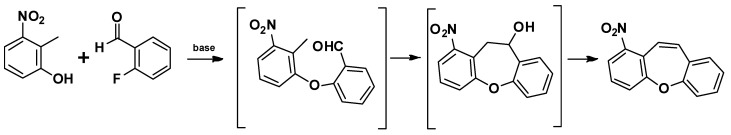
The process involves nucleophilic aromatic substitution followed by Knoevenagel condensation.

An interesting method is a recently developed simple one-pot synthesis of substituted dibenzo[*b*,*f*]oxepines under transition-metal-free conditions (Scheme 8) [73]. This cascade process involves nucleophilic aromatic substitution followed by Knoevenagel condensation. The yield of this process is 77%.

6. Intramolecular McMurry reaction [74]

**Scheme 9:**
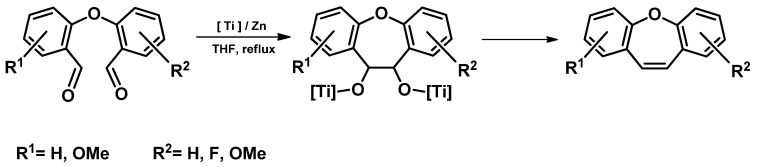
Proposed McMurry reaction course.

Dibenzo[*b*,*f*]oxepines can also be obtained as a result of an intramolecular McMurry reaction. In such, the diaryl ethers are used as the substrates. They can be obtained by the reaction of salicylaldehydes with fluorobenzaldehydes exposed to microwaves. The McMurry reaction for synthesized dibenzo[*b*,*f*]oxepine using TiCl_4_/Zn catalyst in THF has a yield of 53–55%. The synthesis mechanism is still under discussion, but the reaction possibly passes through the intermediate state of metallopinacol formed by the dimerization of ketyl radicals (Scheme 9).

7. Mn(III)-based oxidative radical rearrangement [75]

**Scheme 10:**
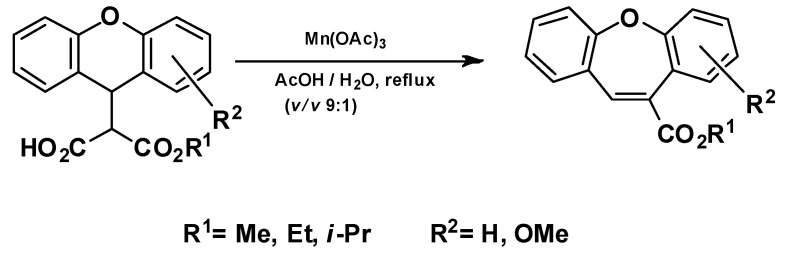
Synthesis of dibenzo[*b*,*f*]oxepines via Mn(III)-based oxidative radical rearrangement.

In 2009, Cong et al. [75] performed a Mn III-based oxidative 1,2-radical rearrangement in order to obtain dibenzo[*b*,*f*]oxepines (Scheme 10). They used a large excess of Mn(OAc)_3_ (4 mol equiv.) in boiling glacial AcOH. The yield of this reaction was in the range of 63%–85% depending on the reaction time, substituents, and additives.

8. Sequential Mizoroki–Heck reaction and Pd-catalyzed etherification [76]

**Scheme 11:**
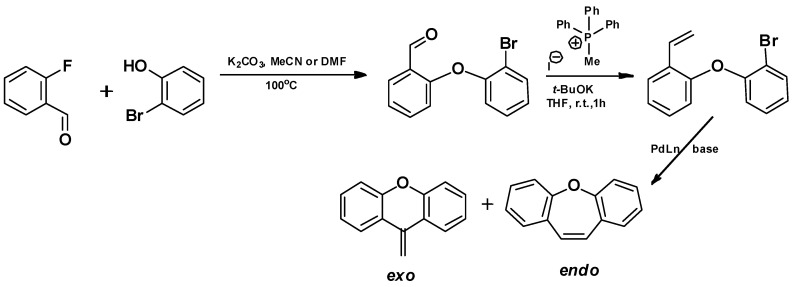
Synthesis dibenzo[*b*,*f*]oxepine in intramolecular Mizoroki–Heck reaction of diaryl ether.

The protocol was based on a palladium-catalyzed intramolecular Mizoroki–Heck reaction of diaryl ethers (Scheme 11). The first step is a nucleophilic aromatic substitution reaction of 2-bromophenol and 2-fluorobenzaldehyde in the presence of potassium carbonate. Next, the obtained product reacts in Wittig olefination using methyl triphenylphosphonium iodide and potassium *tert*-butoxide. Closure of the bromoolefin diaryl ether in the presence of Pd_2_(dba)_3_ tert-butoxide and various phosphine ligands leads to dibenzo[*b*,*f*]oxepine (endo product) and to an exoproduct in the Mizoroki–Heck reaction. The yield of dibenzo[*b*,*f*]oxepine is 59% [76].

9. Oxidative CH bond functionalization and ring expansion with TMSCHN_2_ [77]

**Scheme 12:**
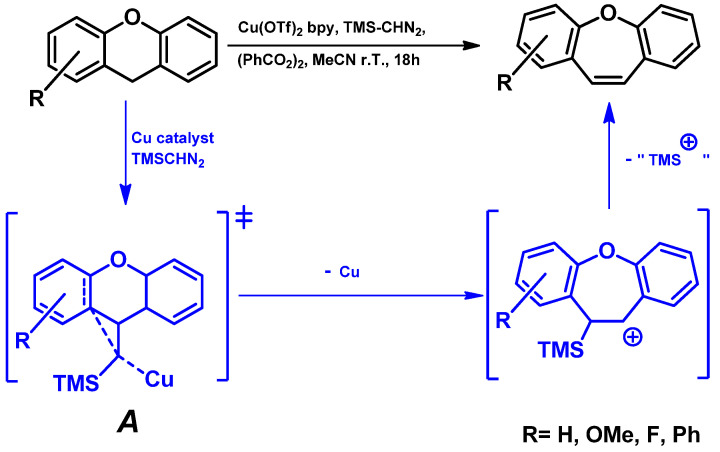
The synthesis of dibenzo[*b*,*f*]oxepines using oxidative CH bond functionalization and ring expansion with the TMS-CHN_2_ method.

The authors [77] proposed a direct C-H functionalization/rearrangement sequence approach (Scheme 12) to obtain dibenzo[*b*,*f*]oxepine. They chose trimethylsilyl diazomethane (TMS-CHN_2_), which might also facilitate or promote the in situ rearrangement to the tricyclic backbone, nonprotic peroxide (PhCO_2_)_2_, a Cu(OTf)_2_ copper catalyst, which is reduced in situ to active Cu^I^ and different ligands such as 2,2′-bipyridine (to improve the properties and stability of the copper catalyst). In summary, the C-H functionalization, insertion, and rearrangement sequence provided the dibenz[*b*,*f*]oxepine scaffold with a moderate 55% yield.

## 5. Active Synthetic Dibenzo[*b*,*f*]oxepin Derivatives

**Figure 4:**
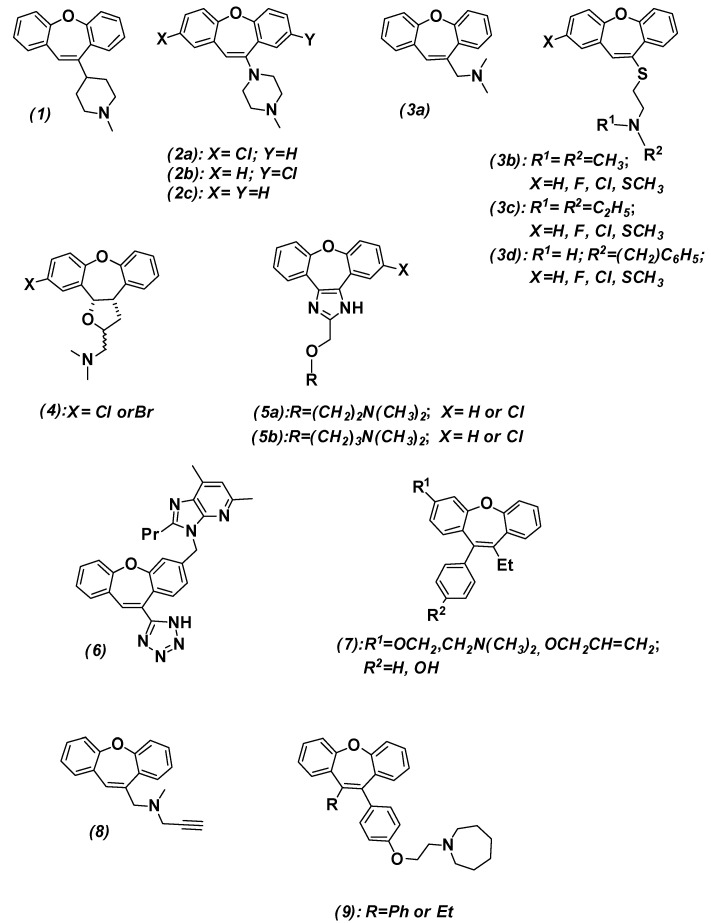
Structure of dibenzo[*b*,*f*]oxepine derivatives (**1**), (**2a**–**2c**), (**3a**–**3d**) type, (**4**), (**5a**,**5b**), (**6**), (**7**), (**8**), and (**9**).

Using the methods presented in the previous paragraph, many dibenzo[*b*,*f*]oxepin derivatives were synthesized and showed biological activity. The antipsychotic properties of these products were studied by the Smith group [78]. They found that among the tested compounds, dibenz[*b*,*f*]oxepine (**1**, Figure 4) has the highest dopamine D-4 receptor activity and is twice as active as clozapine (clozapine is an antipsychotic drug used in the clinic) in blocking the D-4 dopamine receptor. The affinity of 10-(4-methylpiperazino)dibenz[*b*,*f*]oxepine for clozapine binding sites in rat brains was studied [79]. [3H] Clozapine binding in the presence of atropine represents non-muscarinic binding, while binding in the absence of atropine represents muscarinic (cholinergic) binding. It was found that the ratios of the IC_50_ values for dopaminergic to non-muscarinic clozapine binding for (**2a**–**c**) are 9.4 ± 2.0, 7.3 + 2.7, and 4.0 ± 1.3, respectively. In turn, the ratios of the IC_50_ values for dopaminergic to non-muscarinic clozapine binding for clozapine and (**2c**) are the same. Schindler and Blattner [80] studied 10-(dimethylaminomethyl)dibenzo[*b*,*f*]oxepine (**3a,** Figure 4) and its acid addition salts. The research confirmed its adrenolytic and depressant effect on the central nervous system (CNS). It is therefore useful as a sedative, anticonvulsant, and anesthesia enhancer. In 1980, the Kruse group [80,81] detected similar properties for [(alkylamino)ethyl]thio]dibenz[*b*,*f*]oxepines (**3b**–**3d**, Figure 4). Trabanco and Megen’s group [82,83,84] synthesized series *trans* and *cis* tetrahydrodibenzo[*b*,*f*]furo [2,3-d]oxepin derivatives and studied their anxiolytic properties (**4**, Figure 4). The products were evaluated for in vitro affinities for the norepinephrine transporter 5-HT2A and 5-HT2C receptors. This action was also foreseen based on the ED_50_ values obtained in some in vivo assays. The dibenzo[*b*,*f*]oxepine imidazole derivatives (**5a**, **5b**, Figure 4) were recognized as a novel class of tetracyclic compounds with anti-inflammatory activity through the specific inhibition of TNF-a secretion [85]. One of the most interesting examples of the use of dibenzo[*b*,*f*]oxepine is a compound that is an AII (angiotensin II) receptor antagonist (**6**, Figure 4) [86]. Angiotensin II causes an increase in blood pressure, which in turn causes damage to the blood vessels and the heart. AII antagonists bind to angiotensin receptors, thereby preventing angiotensin II itself from binding to its receptor. As a result, this compound may have electrolyte and blood-pressure-regulating properties and antihypertensive properties. The treatment of progressive neurodegenerative diseases such as Parkinson’s and Alzheimer’s diseases [87] with synthetic dibenzo[*b*,*f*]oxepine derivatives is of particular interest. Synthetic dibenzo[*b*,*f]*oxepines as antiestrogenic agents have also been studied [88]. The O-bridged compounds (**7**, Figure 4) were moderately active, and there are no new reports of research in this direction. A very promising derivative with a dibenzo[*b*,*f*]oxepine skeleton is omigapil (TCH346 or CGP3466, **8**, Figure 4), first synthesized at Ciba-Geigy, Basel, Switzerland at the beginning of the 21st century. Santhera Pharmaceuticals has since taken over the production of omigapil and preclinical trials for congenital muscular dystrophy [89]. Notably, omigapil has shown safety in Phase 1 clinical trials for patients with pediatric and adolescent congenital muscular dystrophy (CMD) [89,90,91]. Recent studies with omigapil in rats with traumatic brain injury (TBI), which is the largest non-genetic-, non-aging-related risk factor for Alzheimer’s disease (AD), have shown that TCH346 reduces the level of high-level Tau protein and protects mice from neurodegeneration and AD [92]. Moreover, the derivatives containing the azepane ring presented in Figure 4 (**9**, Figure 4) show a strong inhibitory effect on the proliferation of breast cancer cells of the MDA-MB-231 line [93]. In addition, analgesic [94,95] and antidiabetic [96] properties have been found for dibenzo[*b*,*f*]oxepine derivatives, as well as insecticidal activities [97].

## 6. Potential of Dibenzo[*b*,*f*]oxepines as Microtubule Inhibitors

**Figure 5:**
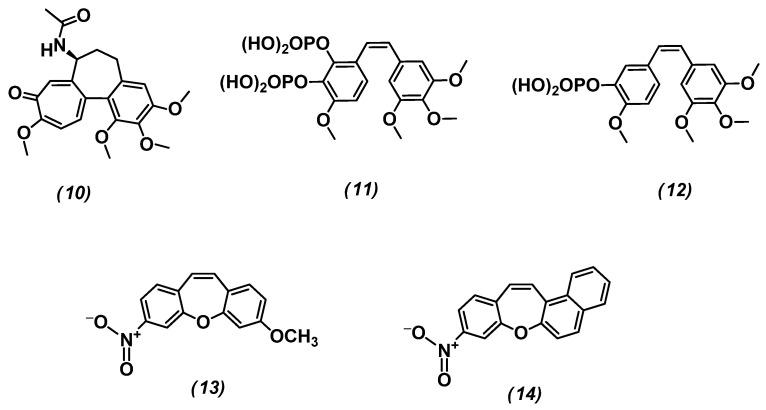
Structue of colchicine (**10**), combretastatin CA1P (**11**), combretastatin CA4P (**12**), and dibenzo[*b*,*f*]oxepines (**13**,**14**).

Notably, a very important new application of dibenzo[*b*,*f*]oxepines is targeting the polymerization/depolymerization of microtubules. Microtubules are fibers that are part of the cytoskeleton. They are composed of α and β-tubulin heterodimers. They form important structures from the point of view of cell division—such as karyokinetic spindles [98]. They are responsible for the alignment of the chromosome lines on the metaphase plate and their precise segregation to the opposite poles during the anaphase of cell division. Thanks to this, when mitosis occurs correctly, the same amount of genetic material goes to the daughter cells. There are chemical compounds that bind to the structural unit of the protein—such as tubulin. Some of them are active substances in anticancer drugs that disrupt the dynamics of microtubules (the balance of their shortening/extension). This phenomenon causes cancer cells to undergo apoptosis [99]. The chemical molecules interact through at least six binding sites: the laulimalide/peloruside binding site, taxane (including paclitaxel, docetaxel, and epothilones), vinca (vinblastine, vincristine, vinorelbine), pironetin, maytansine, and colchicine [100]. Of particular interest from the point of view of anticancer therapies is the colchicine (**10**, Figure 5) binding site. This substance effectively inhibits mitosis by inducing the depolymerization of microtubules, and cancer cells, due to the faster rate of cell division, are more susceptible to its poisoning. However, it is a substance with a low therapeutic index and is also very toxic to healthy cells. It causes, e.g., neutropenia (decrease in the number of neutrophils), gastrointestinal disorders, bone marrow damage, and anemia [101,102]. For this reason, structurally similar substances interacting with the colchicine binding site are sought. Stilbenoids (combretastatins CA1P-**11** and CA4P-**12**, Figure 5) and dibenzo[*b*,*f*]oxepines, which contain the (*Z*)-stilbene motif in their skeleton, show great potential [103]. Several derivatives of these compounds have been studied in the literature, e.g., **13** and **14** (Figure 5). Using molecular modeling, their ability to interact with tubulin was analyzed. The analysis shows that both compounds bind to the colchicine binding site through hydrophobic interactions stabilized by hydrogen bonds. On the other hand, in vitro studies on cell cultures led to the conclusion that 9-nitrobenzo[*b*]naphtho [1,2*-f*]oxepine (**14**) has strong cytotoxic properties, as it does not affect cancer cells selectively, but it also induces apoptosis of “healthy” cells. For this reason, methods are sought to enable “targeted therapies” that selectively affect cancer cells.

**Figure 6:**
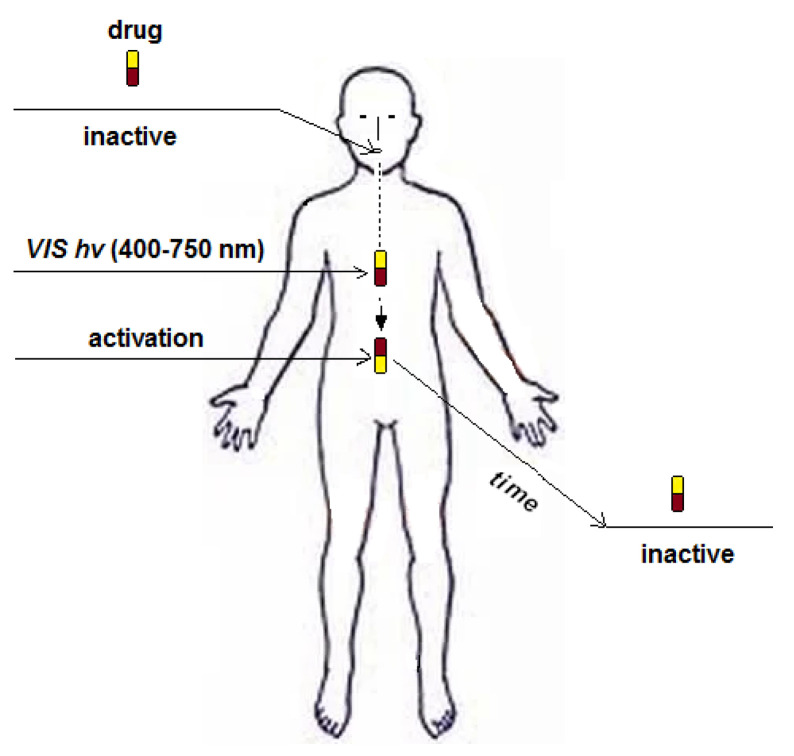
Idea of photopharmacology.

Such a new, but very promising, therapy is photopharmacology (Figure 6) [104,105,106,107,108].

**Figure 7:**
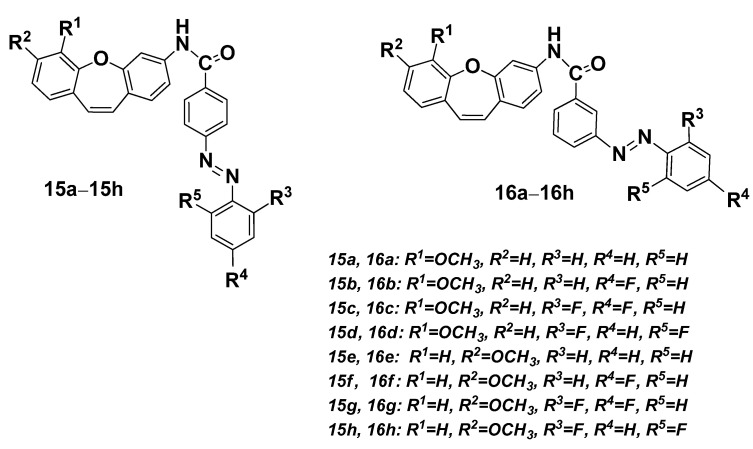
Structure of hybrid dibenzo[*b*,*f*]oxepines with azofluorostilbenes (**15a**–**15h**) and (**16a**–**16h**).

The scope of photopharmacology is the design, synthesis, research, and use of drugs whose activity can be controlled by light. Photopharmacology is a way to solve the activity of a therapeutic off-target (introduction of the drug to the target only after light activation) and a way to avoid side effects. This is possible by controlling the activity of the therapeutic agent from outside the body (Figure 6). Using this therapy, it will be possible to conduct a selective photo-controlled interaction of the drug with a molecular target located in the human body. Molecular switches are molecules that can be reversibly switched between at least two thermodynamically stable states. The pharmacological agents used in photopharmacology are bioactive molecules capable of changing their state, and, thus, their structures, under the influence of light-photochromic molecular switches. Azo compounds are one of the most numerous and most widely studied classes of photochromic molecular switches. They can exist in two isomeric states—*E*/*Z*—with *E* being on average about 10 kcal/mol more stable than *Z*. Derivatives of azobenzene are the most popular and most typically used photochromic molecular switches in biomedical applications. This is due, among other reasons, to their easy synthesis, relatively high photostationary states and quantum efficiency, fast photoisomerization, and low photo-quenching coefficient. It should be emphasized that photochromic switches with an azo group absorb light in the range from 650 nm to 900 nm (visible light range 380–750 nm—harmless), which means that light is not absorbed by water and hemoglobin and can penetrate deeper tissue layers. The combination of the aspects of microtubule polymerization inhibition and electromagnetic radiation activation of compounds containing azo bonds and a dibenzo[*b*,*f*]oxepine backbone with methoxy groups was the subject of research by Borys et al. [109,110]. Firstly, their studies focused on the synthesis of bioactive compounds interacting with tubulin (protein-building microtubules performing the function of the cytoskeleton) and assessing their usefulness in anticancer therapy. Secondly, their research aimed to determine the ability of the newly obtained azo derivatives of methoxydibenzo[*b*,*f*]oxepine to act as molecular switches in the system with tubulin and the way to activate them under the influence of harmless visible light (Figure 7).

In silico studies showed that after combining dibenzo[*b*,*f*]oxepines with an *azo* switch, the molecules interact with the colchicine binding site in tubulin with a part of the dibenzo[*b*,*f*]oxepine, in a part of the azo switch, or both at the same time. Based on the *UV-VIS* spectra, it was found that compounds switch in the visible part of the spectrum and, therefore, these derivatives have the potential to be used in photopharmacology.

To summarize, in Table 2, synthesized dibenzo[*b*,*f*]oxepine derivatives that exhibit biological activity or that have such potential are presented.

## 7. The Perspective for Applications of Dibenzo[*b*,*f*]oxepine Derivatives

In conclusion, in this short review, we discussed the use of synthetic and natural derivatives of dibenzo[*b*,*f*]oxepin. We have shown the use of these compounds mainly in medicine. Several directions can be listed for further research on dibenzo[*b*,*f]*oxepines, searching for new, not-yet-isolated compounds (naturally occurring dibenzo[*b*,*f*]oxepins) from plants that have a healing effect. Omigapil, which exhibits dose-dependent inhibition of HIV, dengue virus, and Zika virus [111], appears to have a high potential for medical use and, as previously mentioned, may alleviate symptoms of congenital muscular dystrophy. Dibenzo[*b*,*f*]oxepines also have the potential as microtubule polymerization inhibitors. The use of combinations of dibenzo[*b*,*f*]oxepines with molecular switches for applications in photopharmacology is another way of using these compounds. The activity of dibenzo[*b*,*f*]oxepines should also be checked, as they are small molecules binding to RNAs implicated in infectious disease (for PreQ(1) riboswitches), whose structure is very similar to the structure of 2-(dibenzo[*b*,*d*]furan-2-yloxy)ethanamine and which have recently been discovered [112,113].

## Figures and Tables

**Table 1:** The XLogP3-AA, TPSA, and hydrogen bond donor/acceptor count parameters for extracted dibenzo[*b*,*f*]oxepines from plants. The data were taken from [45,46]. Norsecocularidine, 4-hydroxysecosarcocapnine, Artocarpol A, Artocarpol G, and Artocarpol I are derived from plants, but there is no information about the parameters listed below.

Entry	Name of Compound	XLogP3-AA	TPSA [Å²]	Hydrogen Bond Donor Count	Hydrogen Bond Acceptor Count
1	Pacharin	3.6	58.9	2	4
2	Bauhiniastatin 1	2.5	72.8	1	5
3	Bauhiniastatin 2	3.5	68.2	2	5
4	Bauhiniastatin 3	3.5	68.2	2	5
5	Bauhiniastatin 4	3.6	58.9	2	4
6	Bauhinoxepin A	4.5	58.9	2	4
7	Bauhinoxepin B	5.5	58.9	2	4
8	Yagonine	2.6	74.3	0	6
9	Aristoyagonine	2.7	57.2	0	5
10	Secocularidine	3.8	51.2	1	5
11	Secocularine	4.2	40.2	0	5
12	Secosarcocapnine	4.2	40.2	0	5
13	Norsecosarcocapnine	3.7	49	1	5
14	Artocarpol D	5.4	58.9	2	4
15	Artocarpol E	7.9	69.9	3	4
16	Artocarpol F	5.1	77.4	2	6
17	Tournefolic acid B	2.8	107	4	6
18	Tournefolic acid B methyl ester	3.2	96.2	3	6
19	Tournefolic acid B ethyl ester	3.5	96.2	3	6
20	Combretastatin A-4	3.7	57.2	1	5

**Table 2:** Synthesized compounds exhibiting biological activity.

Compound	Pharmacological Activities	Figure on Which the Structure Was Placed
**1**	antipsychotic properties	Figure 4
**2a**–**c**	antipsychotic properties	Figure 4
**3a**–**d**	sedative, anticonvulsant, and anesthetic-enhancing properties	Figure 4
**4**	anxiolytic properties	Figure 4
**5a,b**	anti-inflammatory activity	Figure 4
**6**	AII (angiotensin II) receptor antagonist	Figure 4
**7**	antiestrogenic agent (moderately active)	Figure 4
**8**	phase 1 clinical trials with pediatric and adolescent congenital muscular dystrophy; reduce the level of high-level Tau protein	Figure 4
**9**	strong inhibitory effect on the proliferation of breast cancer cells of the MDA-MB-231 line	Figure 4
**13**	the strongest cytotoxic effect against HeLa and U87 cancerous cells lines	Figure 5
**14**	the strongest cytotoxic effect against HeLa and U87 cancerous cells lines	Figure 5
**15a**–**h**	In silico studies showed that compounds **15a**–**h** and **16a**–**h** interact with the colchicine binding site in tubulin with a part of the dibenzo[*b*,*f*]oxepine, in a part of the azo switch, or both at the same time.	Figure 7
**16a**–**h**		Figure 7

## Data Availability

Not applicable.
